# The Human Adenovirus 2 Transcriptome: an Amazing Complexity of Alternatively Spliced mRNAs

**DOI:** 10.1128/JVI.01869-20

**Published:** 2021-01-28

**Authors:** Amanda Westergren Jakobsson, Bo Segerman, Ola Wallerman, Sara Bergström Lind, Hongxing Zhao, Carl-Johan Rubin, Ulf Pettersson, Göran Akusjärvi

**Affiliations:** a Department of Medical Biochemistry and Microbiology, Uppsala University, Uppsala, Sweden; b Department of Immunology, Genetics and Pathology, Uppsala University, Uppsala, Sweden; c Department of Microbiology, National Veterinary Institute, Uppsala, Sweden; d Department of Chemistry–BMC, Analytical Chemistry, Uppsala University, Uppsala, Sweden; Cornell University

**Keywords:** Oxford Nanopore sequencing, adenovirus, alternative splicing

## Abstract

We used the Nanopore long-read sequencing platform to demonstrate how amazingly complex the human adenovirus type 2 (Ad2) transcriptome is, with a flexible splicing machinery producing a range of novel mRNAs from both the early and late transcription units. In total, we report more than 900 alternatively spliced mRNAs produced from the Ad2 transcriptome, more than 850 of which are novel mRNAs. A surprising finding was that more than 50% of all E1A transcripts extended upstream of the previously defined transcriptional start site. The novel start sites mapped close to the inverted terminal repeat (ITR) and within the E1A enhancer region. We speculate that novel promoter- or enhancer-driven transcription, so-called eRNA transcription, is responsible for producing these novel mRNAs. Their existence was verified by a peptide in the Ad2 proteome that was unique to the E1A ITR mRNA. Although we show a high complexity of alternative splicing from most early and late regions, the E3 region was by far the most complex when expressed at late times of infection. More than 400 alternatively spliced mRNAs were observed in this region alone. These mRNAs included extended L4 mRNAs containing E3 and L5 sequences and readthrough mRNAs combining E3 and L5 sequences. Our findings demonstrate that the virus has a remarkable capacity to produce novel exon combinations, which gives the virus an evolutionary advantage, i.e., the ability to change the gene expression repertoire and protein production in an evolving environment.

**IMPORTANCE** Work in the adenovirus system led to the groundbreaking discovery of RNA splicing and alternative RNA splicing in 1977. These mechanisms are essential in mammalian evolution, as they increase the coding capacity of a genome. Here, we used a long-read sequencing technology to characterize the complexity of human adenovirus pre-mRNA splicing in detail. It is mind-boggling that the viral genome, which houses only around 36,000 bp, not being much larger than a single cellular gene, generates more than 900 alternatively spliced mRNAs. Recently, adenoviruses have been used as the backbone in several promising severe acute respiratory syndrome coronavirus 2 (SARS-CoV-2) vaccines. Further improvement of adenovirus-based vaccines demands that the virus be modified into an innocent carrier of foreign genes. This requires a full understanding of the components that govern adenovirus replication and gene expression.

## Abstract (precis)

Work in the adenovirus system led to the groundbreaking discovery of RNA splicing and alternative RNA splicing in 1977. These mechanisms are essential in mammalian evolution, as they increase the coding capacity of a genome.

## INTRODUCTION

The split-gene concept, RNA splicing, and alternative RNA splicing were all discovered in the human adenovirus type (Ad2) system almost 45 years ago (1, 2). In two seminal papers published at the end of the 1970s, Chow and collaborators made an exhaustive electron microscopic characterization of the alternatively spliced mRNA expressed from the Ad2 genome (3, 4). Subsequent cDNA sequencing efforts have characterized the majority of the splice sites at the nucleotide sequence level (reviewed in reference 5). This electron microscopic map has passed the test of time with surprising accuracy, and very little has changed over the last 40 years. The mRNAs expressed from the E1A and E1B regions have been characterized in more detail, and the E2B (6) and the L4 promoter (7) transcripts have been added to the human adenovirus map. Moreover, a novel adenovirus gene, the U gene (8), which has its own promoter, was discovered. Third-generation sequencing has opened new possibilities for characterizing transcriptomes based on the structures of the full-length transcripts. Here, we used the Oxford Nanopore Technologies long-read sequencing platform to unravel the complexity of the Ad2 transcriptome. The result verifies the data of Chow et al. and extends them by showing an amazing complexity of alternatively spliced mRNAs that are expressed during an Ad2 infection.

**FIG 1:**
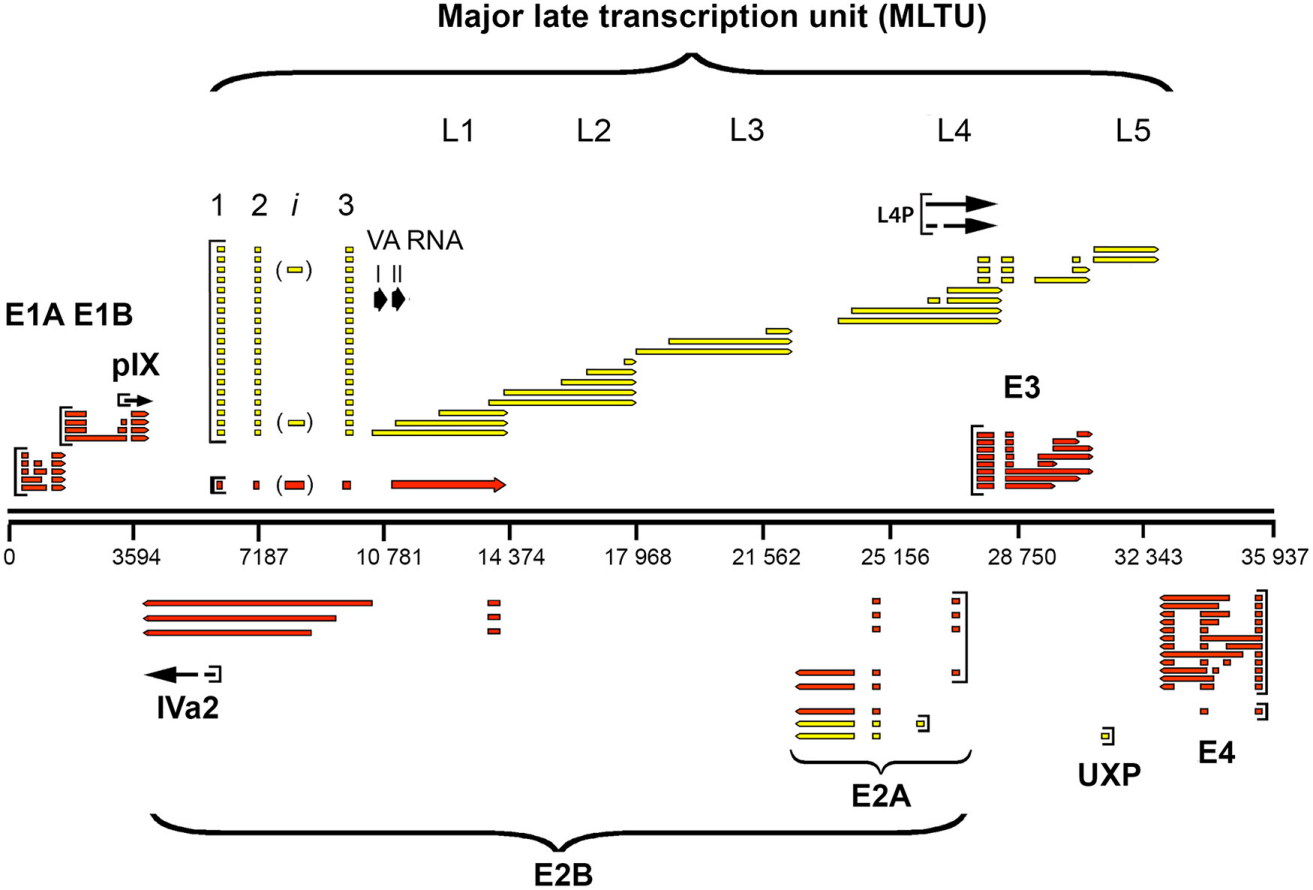
The Ad2 transcriptome before Nanopore sequencing. Schematic view of adenovirus mRNAs expressed from the rightward- and leftward-transcribed strands. The mRNAs are color coded based on time of expression: early transcripts are shown in red, late mRNAs are in yellow, and mRNAs expressed at intermediate times of infection are represented with black arrows.

The adenovirus genome is compact, with essentially no surplus nucleotides that are not transcribed or that have regulatory functions (reviewed in reference 5). In general, adenovirus genes contain few introns compared to cellular genes. Most Ad2 mRNAs mature by removing one to three introns. The extreme example is the pIX mRNA, which is an unspliced mRNA (9). Cellular genes have, on average, eight introns. Previous studies have shown that adenoviral introns, in contrast to introns in cellular genes, rarely interrupt the protein-coding portion of the genes (the notable exception is E1A). In adenovirus, introns are typically positioned at the 5′ end, although some of them occur in the 3′ untranslated region (UTR) of the mRNA. This is in striking contrast to cellular genes, where introns typically interrupt the coding portion of the gene. Both strands of the approximately 36,000-bp viral DNA genome are transcribed and encode proteins. The viral genome is subdivided into multiple transcription units that are expressed at different phases of the virus life cycle: the early, intermediate, and late units (Fig. 1). The early genes include early region 1A (E1A), E1B, E2A, E2B, E3, and E4. They are predominantly transcribed during the first 24 h of infection and have functions important for promoting viral transcription and replication and avoiding the host cell immune response. The main functions of the intermediate and late genes are to code for structural components of the viral capsid and regulatory factors, facilitating viral gene expression and virion assembly (for a general review of adenoviruses, see reference 10). The functions of different genes are described briefly in Results.

In addition to the above-mentioned protein-encoding genes, which are transcribed by RNA polymerase II, adenovirus encodes two extremely abundant RNA polymerase III products, the so-called virus-associated RNAI (VA RNAI) and VA RNAII. VA RNAI is crucial for the viral infection by inhibiting the interferon-induced protein kinase R (PKR). The VA RNAs are also processed by the microRNA (miRNA) machinery to viral miRNAs (mivaRNAs) (reviewed in reference 11).

## RESULTS

### Experimental approach.

Human primary lung fibroblast (IMR-90) cells were used in these experiments. The kinetics of infection in IMR90 cells is slower than in HeLa cells (12), with viral DNA replication initiating around 24 h postinfection (hpi). IMR90 cells were infected with Ad2 and cytoplasmic RNA isolated at 12, 24, and 36 hpi. The full transcriptome of Ad2 was studied using Oxford Nanopore Technologies (ONT) long-read sequencing technology, applying two approaches. In the first, the mRNAs were reverse transcribed to cDNAs, subjected to PCR-based amplification, and then subjected to ONT sequencing. Alternatively, the mRNA pool was subjected to an ONT sequencing-based direct RNA sequencing strategy. This protocol avoids potential artifacts generated during the reverse transcription and PCR-based steps of amplification. The drawback of the direct RNA sequencing approach is that the sequence depth is much reduced compared to that seen with the cDNA sequencing protocol. To verify the accuracy of the mRNA structures, we used a triple-verification strategy (see Materials and Methods). Since we used a rigorous verification protocol, we could afford to employ a cutoff value as low as five total reads for the mRNAs presented in the figures. However, we present all triple-verified mRNA structures in Table S1 in the supplemental material.

### Handling of transcriptional start and polyadenylation sites.

Full-length cDNA-PCR-based sequencing relies on an efficient reverse transcription reaction, and the RNA direct sequencing relies on a highly processive Nanopore motor protein. These enzymes were not expected to function perfectly. Therefore, and because of the reduced raw base-calling accuracy of ONT sequencing, we were not expecting to be able to map transcriptional start sites and polyadenylation sites with nucleotide precision, only to identify regions involved in transcription initiation and poly(A) site usage. On a grander scale, the poly(A) sites are all located at the expected positions. However, a close examination of the poly(A) sites shows that all have heterogeneity at the 3′ ends. We refrain from a more in-depth discussion of transcriptional start sites and poly(A) sites, since these have to be experimentally verified before any conclusions can be drawn.

### Resequencing of the Ad2 genome.

To increase the accuracy of spliced-mRNA detection, we resequenced the genome of the Ad2 isolate used in this study. Six point mutations (A264A, G1134C, G4573A, G5043C, C5751T, and G17964C) were observed compared to the published human Ad2 reference sequence (GenBank no. J01917.1) (13). No heterogeneity in the population was observed at the DNA level, and the mutations were not located at any of the described splice sites. These mutations may reflect sequencing errors in the reference sequence and/or mutations that have accumulated during cultivation of the virus in our laboratory during the last 40 years.

### Early regions 1A and 1B.

**FIG 2:**
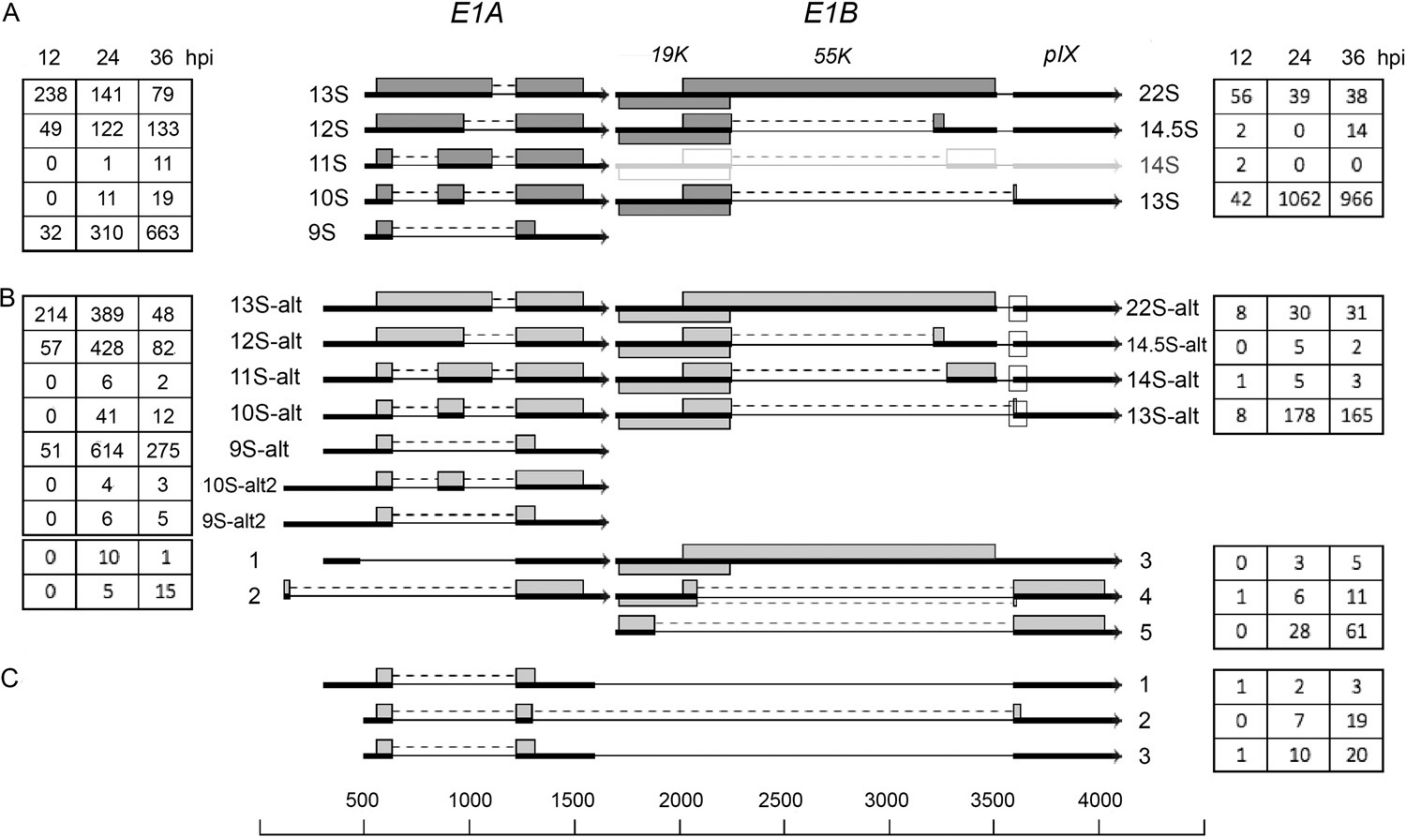
The E1A and E1B family of mRNAs. (A) Previously characterized mRNAs from the E1 region. The characterized E1B 14S mRNA (16) is shown in light gray, since the number of reads was below our cutoff. (B) Novel E1 mRNAs identified. (C) Structure of the most abundant E1A-E1B fusion mRNAs. The numbers of reads identified at 12, 24, and 36 hpi are shown within the boxes on the left (E1A) or on the right (E1B). The squares mark the E1B mRNAs using the alternative minor 3,591 3′ splice site. Thick lines denotes exons, thin lines introns. Gray boxes indicate the proposed protein(s) expressed from respective mRNA. For a complete summary of the triple-verified E1 mRNAs, see Table S1.

Previous studies identified five alternatively spliced mRNAs in the E1A unit with a transcriptional start site mapping to position 498: 13S (encoding the E1A-289R transcriptional regulatory protein), 12S (encoding the E1A-243R cell cycle regulator), and 11S, 10S, and 9S, of hitherto-unknown function(s) (Fig. 2A) (reviewed in reference 10). Interestingly, the majority of the E1A mRNAs we detected showed an extended 5′ end, indicating the existence of upstream promoters driving E1A mRNA expression. The most abundant of these were the mRNAs with heterogeneous 5′ ends mapping to the viral DNA packaging domain (reviewed in reference 14), the so-called packaging A repeats 1 to 7 (positions 239 to 374) (Fig. 2B). This family of mRNAs combined was as abundant as the classical E1A mRNAs, with a transcriptional start site at position 498. Their introns are identical to those present in the classical 9S to 13S mRNAs (Fig. 2A). Since the extended 5′ end lacks an AUG initiation codon, they would be predicted to encode proteins identical to the classical E1A proteins.

A putative promoter within the inverted terminal repeat (ITR) produces three low-abundance mRNAs (10S-alt2, 9S-alt2, and transcript 2) (Fig. 2B), predominantly at the late stage of infection. Transcript 2 has a novel 5′ splice site at position 146 that connects to the common E1A 3′ splice site at position 1,226. The predicted protein expressed from this mRNA would connect a 9-amino-acid peptide in frame to the common C-terminal exons present in all E1A proteins, except the 9S protein (Fig. 2B). Interestingly, our mass spectrometry analysis of proteins expressed during an Ad2 infection (15) identified a unique peptide that most likely is derived from this predicted protein (see Discussion). Transcript 2 is expressed at levels similar to those of the E1A 11S mRNA. The 9S-alt2 and 10S-alt2 mRNAs are unlikely to be translated to the 9S and 10S proteins, since the longer 5′ UTR contains multiple potential AUG triplets.

The classical E1B 13S and 22S mRNAs, containing one intron and the minor 14S and 14.5S mRNAs (16), each containing two introns, comes in two spliced variants. The previously characterized 3′ splice site at position 3588, which is common to all E1B mRNAs, has an alternative minor 3′ splice site at position 3591 (depicted by a square in Fig. 2B). All four mRNAs come in these two variants. Since this alternative splicing event is in the 3′ UTR of all mRNAs except the 13S mRNA, it does not cause any change in protein expression. The shortened 55,000-molecular-weight protein (55K protein) expressed from the minor 13S-alt mRNA, using the 3,591 3′ splice site, would lose a glutamine compared to the protein expressed from the major 13S mRNA.

We also detected a low abundance of an unspliced E1B mRNA (Fig. 2B, transcript 3). In addition, two novel 5′ splice sites in the E1B first exon were also identified (transcripts 4 and 5; Fig. 2B). In both mRNAs, the predicted E1B proteins expressed would be fusion proteins with the late pIX translational reading frame (transcript 4, with the 55K open reading frame [ORF] fused to pIX, and transcript 5, with the 19K ORF in frame with pIX).

Readthrough transcripts of E1A and E1B have previously been reported (17). Here, we identified a large class of low-abundance E1A and E1B fusion transcripts (Table S1). Only three of these mRNAs exceeded the threshold imposed here (Fig. 2C). In all three mRNAs, which were most abundantly expressed at 36 hpi, an E1A 9S mRNA connected through two different 5′ splice sites in the E1A last exon to the E1B 3588 3′ splice site. The encoded protein would be the E1A 9S protein or an E1A 9S protein with an alternative carboxy terminus derived from the E1B last exon (Fig. 2C, transcript 2).

### Early regions 2A and 2B and UXP.

The E2 transcription unit (Fig. 1) encodes the viral proteins required for genome replication: the E2A-72K single-stranded DNA binding protein, the E2B preterminal protein, and the Ad DNA polymerase (reviewed in reference 10). In addition, an upstream promoter located within the L5 protein-coding sequence produces a leftward-transcribed mRNA that encodes the U exon protein (UXP), which associates with Ad replication centers (8).

**FIG 3:**
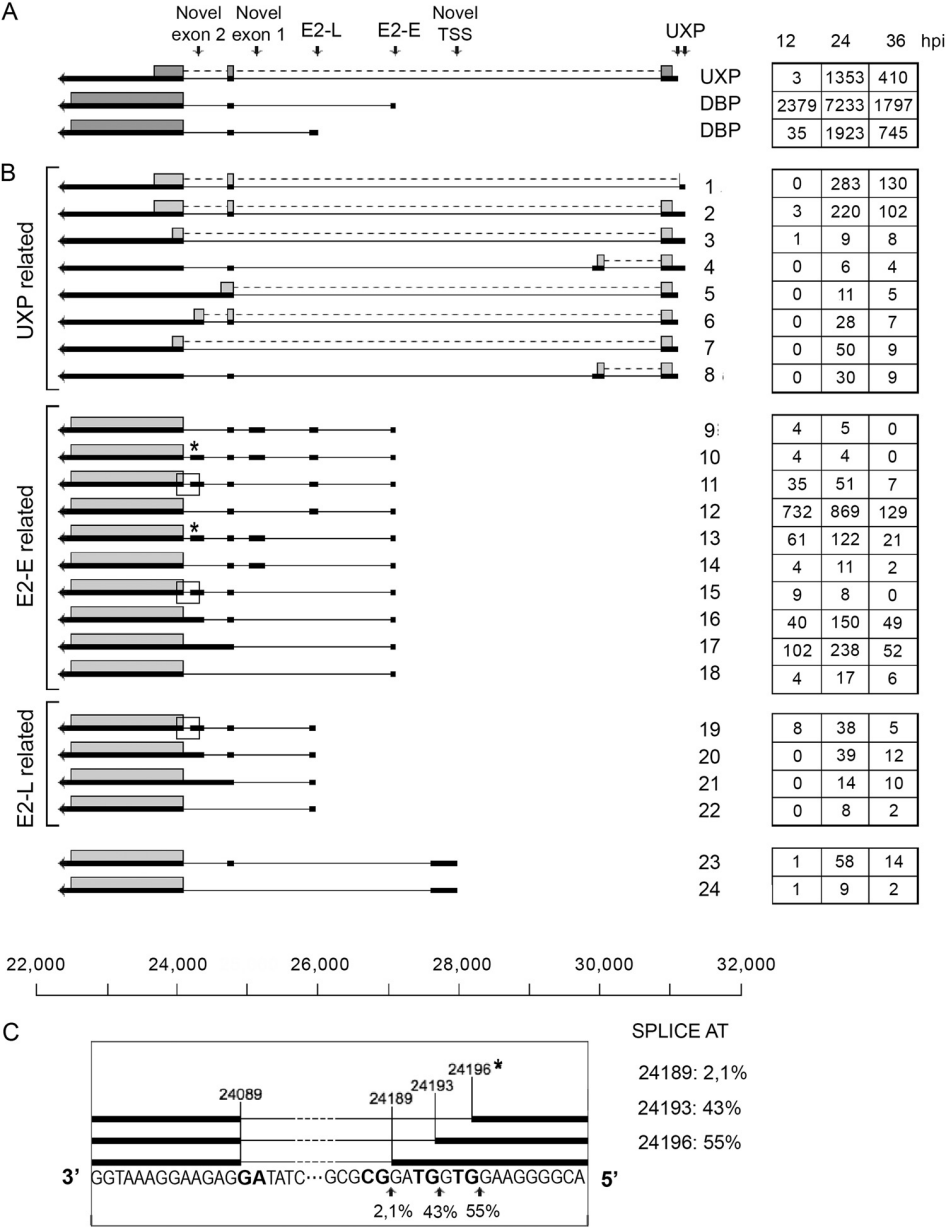
The E2A and UXP family of mRNAs. (A) Previously characterized E2A and UXP mRNAs. (B) Novel E2A and UXP mRNAs grouped into mRNAs with the same transcriptional start site. The numbers of reads identified at 12, 24, and 36 hpi are shown in boxes on the right. Thick lines denote exons, thin lines introns. Gray boxes indicate the proposed proteins expressed from respective mRNA. The asterisk and the boxed areas in panel B indicate a microheterogeneity in 5′ splice site usage at novel exon 2, which is shown expanded in panel C. TSS, transcriptional start site. For a complete summary of the triple-verified E2 and UXP mRNAs, see Table S1.

The complexity of UXP mRNA splicing was much higher than previously described (8) (Fig. 3). As shown in Fig. 3B, deletion of an exon or introduction of a novel exon in combination with alternative splicing of the last exon would result in mRNAs, predicted to produce minor UXP proteins with a truncated C terminus (transcripts 5 to 8). In addition, the sequence result implies the existence of an alternative upstream UXP promoter (transcript 1 to 4). Three of these transcripts have out-of-frame AUG triplets and may or may not encode UXP (transcripts 2 to 4). Transcript 1 uses an upstream 5′ splice site and could theoretically use an alternative AUG start codon that is in frame with the UXP protein and produce a UXP protein with an N-terminal deletion. Collectively, the alternative UXP mRNAs (Fig. 3B) would account for more than 30% of all UXP mRNAs.

The E2 early and E2 late promoters (E2-E and E2-L; Fig. 3) also produce mRNAs with a more complex exon arrangement than previously described for Ad2. As shown in Fig. 3B, 14 novel transcripts were identified (transcripts 9 to 22), which differed from the canonical E2 mRNAs (Fig. 3A). Two novel exons were also discovered that both have potential AUGs that might be used for the synthesis of short peptides. Interestingly, novel exon 2 (Fig. 3B, transcripts 11, 15, and 19) also has heterogeneity with three potential 5′ splice sites, one of which would deviate from the GT-AG rule (Fig. 3C). This minor GC-AG splicing event has been triple verified. Counting the microheterogeneity in novel exon 2 splicing as separate transcripts, we would report a total of 19 new alternatively spliced E2A mRNAs. A novel potential transcriptional start site was observed between the E2-E promoter and the UXP promoter (Fig. 3B, transcripts 23 and 24) that would produce novel low-abundance mRNAs that most likely would encode the DNA-binding protein (DBP). The E2B mRNAs (Fig. 1) were expected to be rare (6), and we found only two full-length E2B mRNAs in our triple-verification protocol.

### Early region 3.

**FIG 4:**
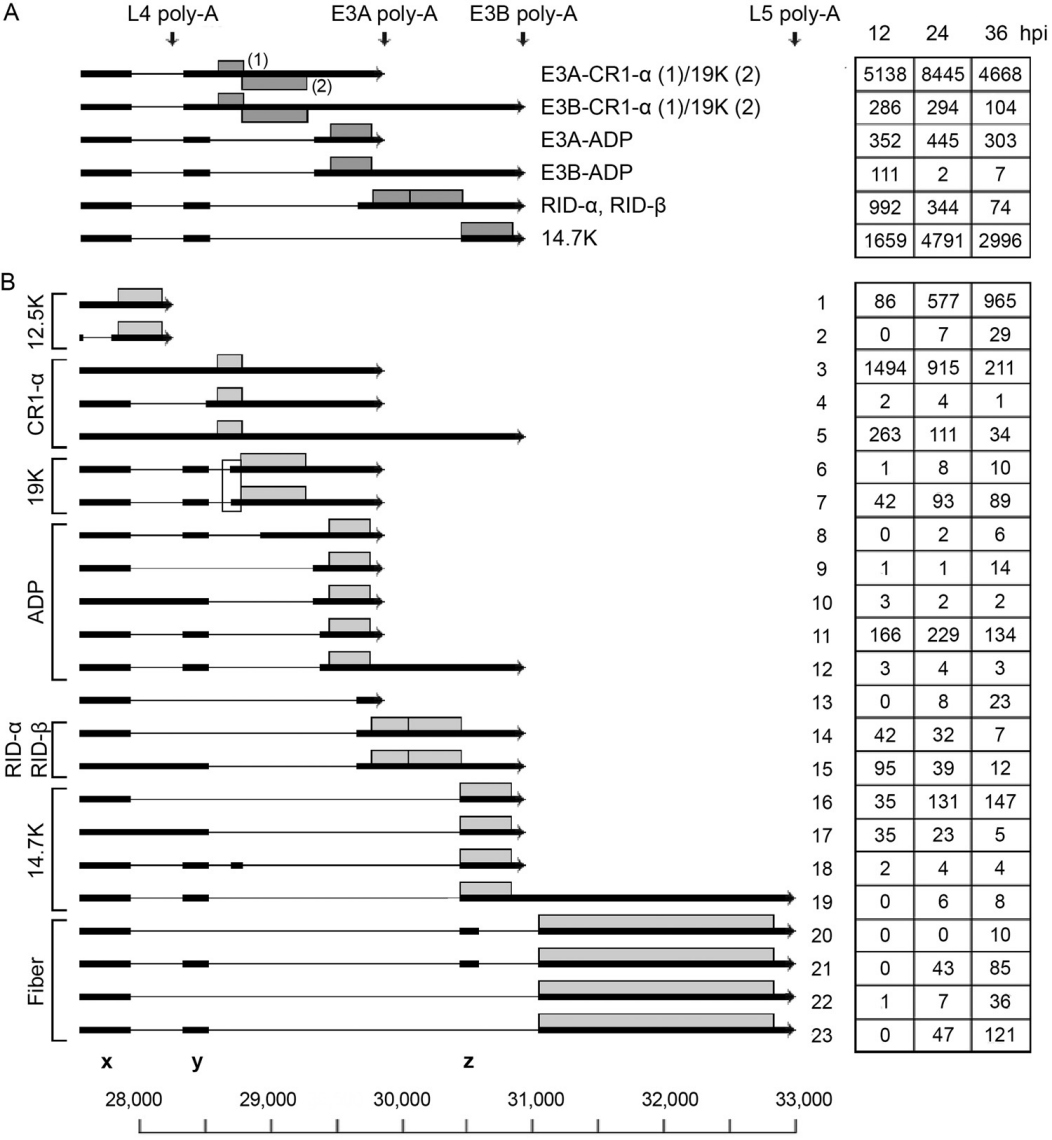
The E3 family of mRNAs. (A) Previously characterized E3 mRNAs with the likely proteins translated shown as gray boxes. (B) Novel E3 mRNAs grouped into mRNAs based on the likely protein encoded (gray boxes). The boxed area shows two gp19K mRNAs that use two alternative 3′ splice sites separated by 16 nucleotides. The number of reads identified at 12, 24 and 36 hpi are shown within boxes on the right. Thick lines denotes exons, thin lines introns. Poly-A, polyadenylation site. For a complete summary of the triple-verified E3 mRNAs, see Table S1.

The E3 transcription unit is embedded in the major late transcription unit (MLTU) (Fig. 1) and encodes proteins that protect the virus from the cellular immune response (reviewed in reference 10). At the early stage of infection, the E3 unit is transcribed from its own promoter at position 27609, whereas at the late stage of infection, E3 mRNA transcription is primarily under the control of the major late promoter (MLP) (see below). E3 has two major poly(A) sites, generating two families of E3 transcripts: the E3A and E3B mRNAs (Fig. 1). A third poly(A) site between E3A and E3B has been observed only with the electron microscope (3). We detect a low abundance of mRNAs that might correspond to this group of poly(A) mRNAs (Table S1). Based on cDNA sequencing, six mRNAs have been described, encoding the characterized E3 proteins (Fig. 4A). Splicing events including splice sites flanking the so-called x, y, and z leaders, which are also spliced to the L5 fiber mRNA (see below), are used extensively in alternative splicing of the E3 mRNAs.

Our analysis demonstrated a more complex usage of poly(A) sites than has previously been shown. Thus, we detected mRNAs initiated at the E3 promoter that use four alternative poly(A) sites (Fig. 4): the L4, E3A, E3B, and L5 fiber poly(A) sites. In Fig. 4B, the mRNAs are grouped to illustrate the variable splicing patterns generating mRNAs that would be predicted to encode the different E3 proteins. The E3-gp19K protein has previously not been assigned to a unique mRNA by cDNA sequencing, although the original electron microscopic study (3) identified a candidate gp19K mRNA. Here, we identified this mRNA and showed that two alternatively spliced mRNAs have the potential to encode the gp19K protein (transcripts 6 and 7; Fig. 4B). They differ by using two alternative 3′ splice sites separated by 16 nucleotides (boxed region in Fig. 4B).

At the later stages of infection, a small portion of the E3 mRNAs extend beyond the E3B poly(A) site and instead use the L5 poly(A) site (transcripts 19 to 23; Fig. 4B), forming a subfamily of E3-L5 mRNAs driven by the E3 promoter. These E3-L5 transcripts differ by using distinct combinations of the x, y, and z leaders. Splicing to the z-leader 3′ splice site in the E3-L5 mRNAs will result in the inclusion of the complete 14.7K reading frame (transcript 19; Fig. 4B). Whether this mRNA is translated to 14.7K, the fiber, or both is an open question.

### Early region 4.

**FIG 5:**
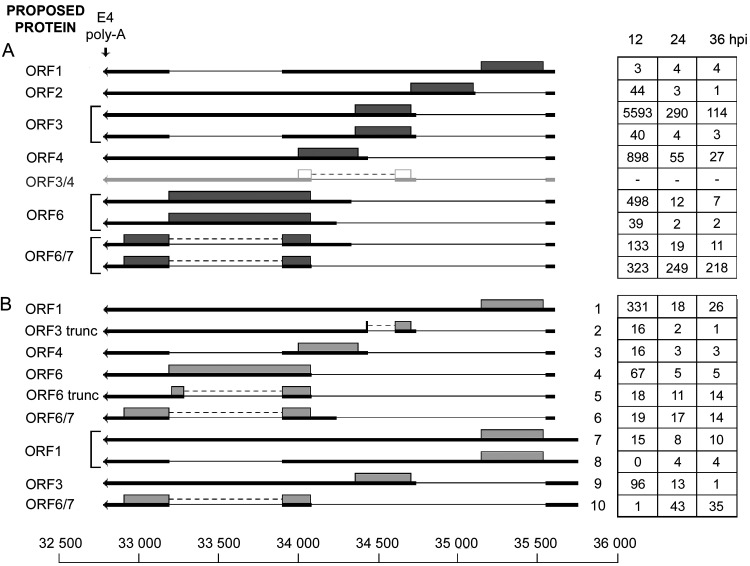
The E4 family of mRNAs. (A) Previously characterized E4 mRNAs, with the likely proteins translated shown as gray boxes. The ORF3/4 mRNA is shown in light gray, since we could not detect reads corresponding to this mRNA in our Nanopore data. (B) Novel E4 mRNAs grouped based on the promoter position. The numbers of reads identified at 12, 24, and 36 hpi are shown in boxes to the right. Thick lines denotes exons, thin lines introns. Gray boxes indicate the proposed proteins expressed from respective mRNA. Poly-A, polyadenylation site. For a complete summary of the triple-verified E4 mRNAs, see Table S1.

The E4 transcription unit (Fig. 5) has been shown to produce a complex set of mRNAs with the potential to encode six different proteins (Fig. 5A). Our analysis confirmed the majority of the identified splice sites in E4 and placed them in the context of full-length transcripts (Fig. 5). Of the 12 mRNAs previously identified (18), nine were confirmed in this study. In addition, we identified two unspliced mRNAs and eight novel splicing combinations (Fig. 5B), which encode the expected proteins, except for transcripts 2 and 5, which encode E4-ORF3 and E4-ORF6 proteins with an in-frame deletion (ORF3 trunc and ORF6 trunc; Fig. 5B). In most of the mRNAs, alternative splicing connects a common 5′ leader exon to different 3′ splice sites, generating the plethora of E4 mRNAs (Fig. 5). In several mRNAs, an intron is excised (positions 33903 to 33193), generating two variant mRNAs encoding the same protein (for example, see the ORF3 mRNAs in Fig. 5A). In the E4-ORF6 mRNA, removal of this internal intron creates the mRNA encoding the E4-ORF6/7 protein (Fig. 5A), which is important for activation of E2 transcription (reviewed in reference 10).

In addition, we observed a cluster of novel mRNAs, which included the introns described above but appeared to initiate transcription from a promoter located upstream of the previously characterized E4 promoter (Fig. 5B, transcripts 6 to 9). This potential promoter is located close to the right-hand ITR and could be the equivalent of the eRNA promoter we detected in the E1A region (Fig. 2B).

### Intermediate transcription units IVa2 and pIX.

IVa2 is a multifunctional protein that has a key function as the ATP-dependent viral DNA packaging protein and also functions as a transcription factor activating the MLP. The pIX protein is primarily a glue protein stabilizing the facets of the virion, but it has also been shown to have the capacity to activate some of the promoters (reviewed in reference 10). It is transcribed from promoters driving transcription in the opposite direction with juxtaposed poly(A) sites (Fig. 1).

**FIG 6:**
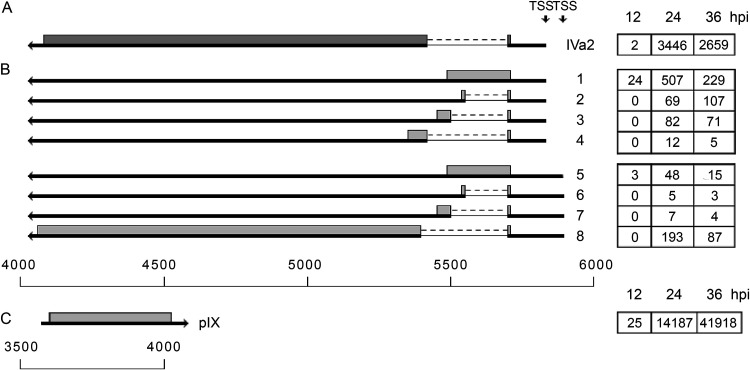
The IVa2 and pIX family of mRNAs. (A) Spliced structure of the previously characterized IVa2 mRNA. (B) Novel IVa2 mRNAs grouped based on three apparent promoter positions. (C) Unspliced pIX mRNA. The numbers of reads identified at 12, 24, and 36 hpi are shown in boxes on the right. Thick lines denotes exons, thin lines introns. Gray boxes indicate the proposed proteins expressed from respective mRNA. TSS, transcriptional start site. For a complete summary of the triple-verified mRNAs from these regions, see Table S1.

The IVa2 transcriptional start site is located in close proximity to the MLP (separated by 212 bp) and drives transcription in the reverse direction to MLP (Fig. 1). This unit expresses an abundant spliced mRNA that has been described previously (Fig. 6A). However, a closer examination of the composition of the alternatively spliced IVa2 mRNAs suggests that an unspliced mRNA exists and that there are four alternative 3′ splice sites used within this unit at a low frequency. These alternative splicing events would generate four alternative proteins that all maintain the first four amino acids from IVa2 (Fig. 6B, transcripts 1 to 4). In addition, essentially the same IVa2 transcripts were generated from a potential IVa2 promoter located approximately 70 bp upstream of the previously characterized (Fig. 6B, transcripts 5 to 8).

The pIX transcription unit is embedded in E1B (Fig. 1 and 2). At late times of infection, the pIX mRNA was the most abundant mRNA detected in our analysis (Fig. 6C). We observed pIX mRNA variants at a low frequency that appear to have bypassed the normal E1B/pIX poly(A) site and terminate at poly(A) sites used by the MLTU mRNAs. However, none of these could be verified by the triple-verification strategy. They were absent from the Nanopore direct RNA sequencing.

### The MLTU.

The MLTU encodes all the structural proteins of the virion except for pIX, which is encoded from its own transcription unit embedded within E1B (Fig. 1). The MLTU also encodes a group of nonstructural proteins that have key functions during the virus life cycle (reviewed in reference 10). Transcription from the MLTU is initiated at the MLP and generates a primary transcript of approximately 28,000 nucleotides that is processed into a large number of cytoplasmic mRNAs. These are grouped into five families (L1 to L5; Fig. 1), where each family consists of multiple alternatively spliced mRNAs with a shared poly(A) site. An important consequence of the processing pathway is that the vast majority of MLTU mRNAs have a common 201-nucleotide tripartite leader sequence at their 5′ end (Fig. 1).

### Tripartite leader splicing.

**FIG 7:**
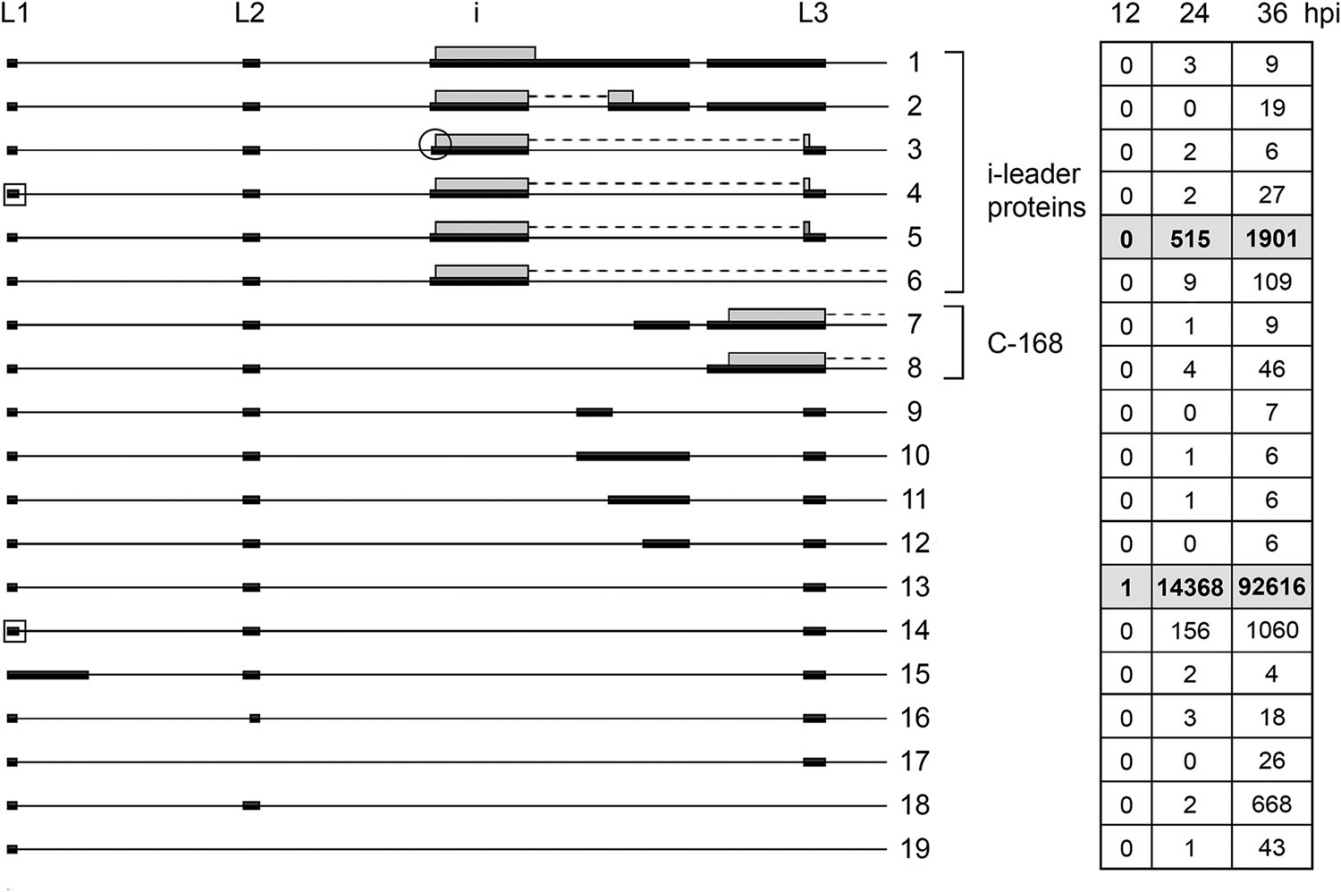
Tripartite leader splicing. Alternative splicing events detected in the tripartite leader region are shown. Transcripts have been organized into groups to highlight the proposed proteins being translated and structural similarities between noncoding RNAs. The numbers of reads identified at 12, 24, and 36 hpi are shown in boxes to the right, with previously described transcripts in bold in shaded boxes. Thick lines denotes exons, thin lines introns. The square box indicates an alternative L1 5′ splice site located 4 nucleotides downstream of the major 5′ splice site. The encircled i-leader 3′ splice site is located 9 nucleotides downstream of the major i-leader 3′ splice site. Gray boxes indicate the proposed i-leader and C-168 proteins expressed from respective mRNA. For a complete summary of the triple-verified transcripts, see Table S1.

Recent work has shown a more complex splicing pattern within the tripartite leader region than was previously thought (19). The tripartite leader, which lacks AUGs, is spliced to different mRNA bodies within the MLTU. It functions as a cap-independent translational enhancer (20). Earlier studies showed that a small fraction of the MLTU mRNAs have an extra 440-nucleotide i-leader exon spliced in between leader 2 and 3 in the tripartite leader (Fig. 7) (3, 21).

A low frequency (approximately 2% of total tripartite leader splicing) of alternative splicing events within the leader region was detected at 36 hpi. Most conspicuous was the complex variability of alternative splicing events between the i leader and leader 3 (Fig. 7). It is noteworthy that this region encodes two proteins. Inclusion of the i leader leads to the synthesis of the 16K i-leader protein (22). In addition, the C-168 protein (23) is most likely translated from transcripts 7 and 8 (Fig. 7), which contain an extended leader 3. Interestingly, both the i-leader and C-168 proteins carry variable C termini. The classical i-leader mRNA (Fig. 7) has a C terminus encoded from leader 3, whereas transcripts 1, 2, and 6 (Fig. 7) generate i-leader proteins with alternative C-terminal amino acids. The i-leader protein translated from transcript 6 and the C-168 protein translated from transcripts 7 and 8 generate proteins with a variable C terminus depending on which late mRNA body the variant leader combinations are spliced to. We have, so far, not detected any specificity of MLTU 3′ splice site usage for these two variant leader splicing events.

### The L1 family of mRNAs.

**FIG 8:**
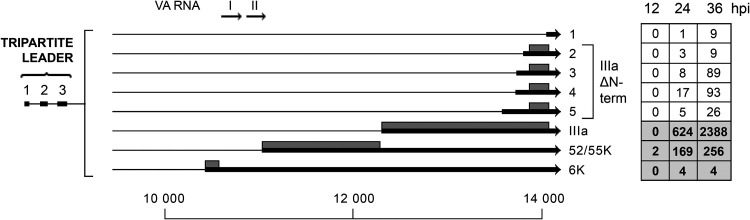
The L1 family of mRNAs. Alternative spliced mRNAs detected from late region 1 are shown. The numbers of reads identified at 12, 24, and 36 hpi are shown in boxes on the right, with previously described transcripts in bold in shaded boxes. Thick lines denotes exons, thin lines introns. Gray boxes indicate the proposed proteins expressed from respective mRNA. For a complete summary of the triple-verified L1 mRNAs, see Table S1.

L1 generates three mRNAs that were previously verified (Fig. 8). The 52/55K protein is an accessory protein in virus genome packaging, and the IIIa protein is a structural protein of the virion (reviewed in reference 10). As expected from previous work (3, 21), the 52/55K mRNA is the first MLTU mRNA to accumulate, being already detectable at 12 hpi (Fig. 8). The predicted 6K protein encoded from the third mRNA has never been detected, although this 3′ splice site has been verified by multiple strategies. Of note, the 3′ splice site for the 6K mRNA is located upstream of the VA RNAI and II genes, making these two regulatory noncoding RNAs part of an mRNA.

Our Nanopore analysis demonstrated the existence of five additional 3′ splice sites within region L1 (Fig. 8). Interestingly, four of these mRNAs would be predicted to encode a 66-amino-acid protein corresponding to the C terminus of the structural IIIa protein. The abundance of these four novel mRNAs is low but significantly higher than that of the characterized 6K mRNA (Fig. 8). In fact, the abundance of transcripts 2 to 5 combined (Fig. 8) is more than 50% of that of the 52/55K mRNA.

### The L2 family of mRNAs.

**FIG 9:**
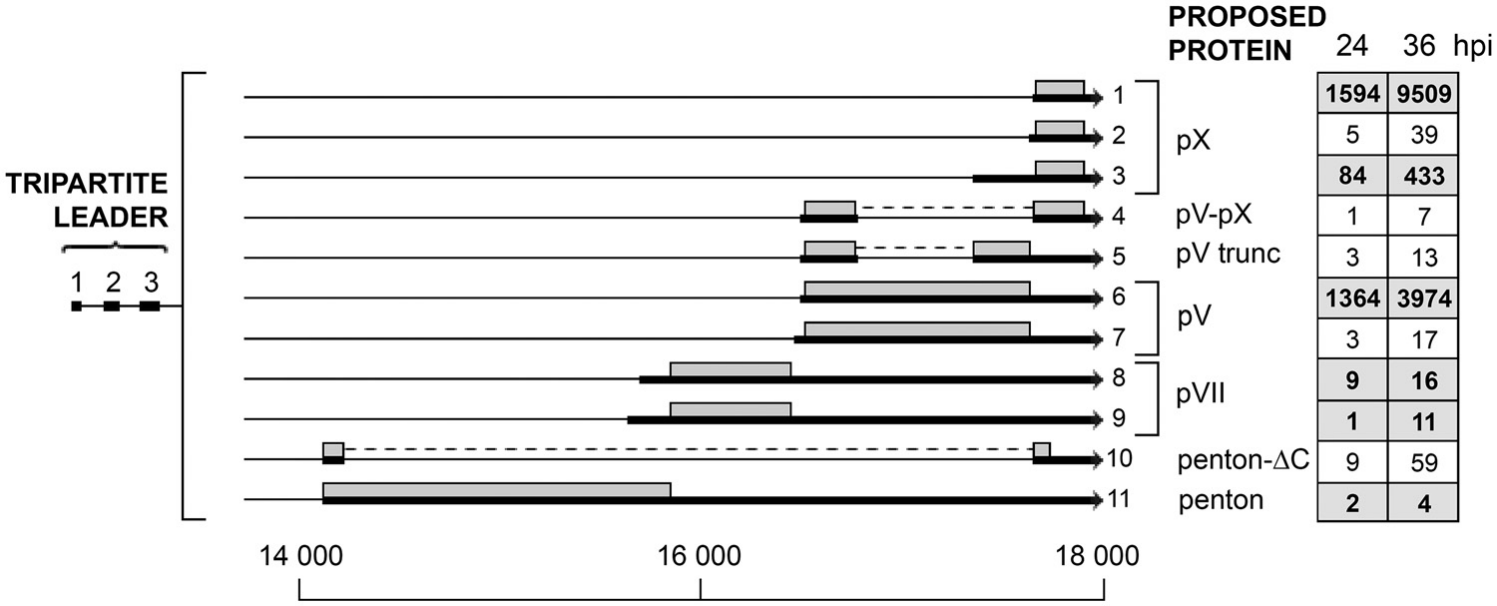
The L2 family of mRNAs. Alternative spliced mRNAs detected from late region 2 are shown. The numbers of reads identified at 24 and 36 hpi are shown in boxes on the right, with previously described transcripts highlighted in bold in shaded boxes. Thick lines denotes exons, thin lines introns. Gray boxes indicate the proposed proteins expressed from respective mRNA. For a complete summary of the triple-verified L2 mRNAs, see Table S1.

The L2 unit encodes the penton base (III) and the three viral core proteins (pVII, pV, and pX) (reviewed in reference 10). The expected mRNAs encoding the classical L2 proteins were all found (Fig. 9). The pV and pX mRNAs were clearly the most abundant in this analysis. We believe that the full-length pVII and penton mRNAs are underrepresented in this analysis, since we noted an unusually large fraction of the penton and pVII transcripts ending prematurely within a broad region of the L2 region.

We observed five novel low-abundance mRNAs from region L2. Two of these represent additional pV and pX mRNAs with a novel 3′ splice site (transcripts 7 and 2; Fig. 9). Also, we observed three mRNAs with internal splicing events predicted to generate a fusion protein between pV-pX and a penton-ΔC protein, and also an internal splicing event generating a truncated pV protein (Fig. 9).

### The L3 family of mRNAs.

**FIG 10:**
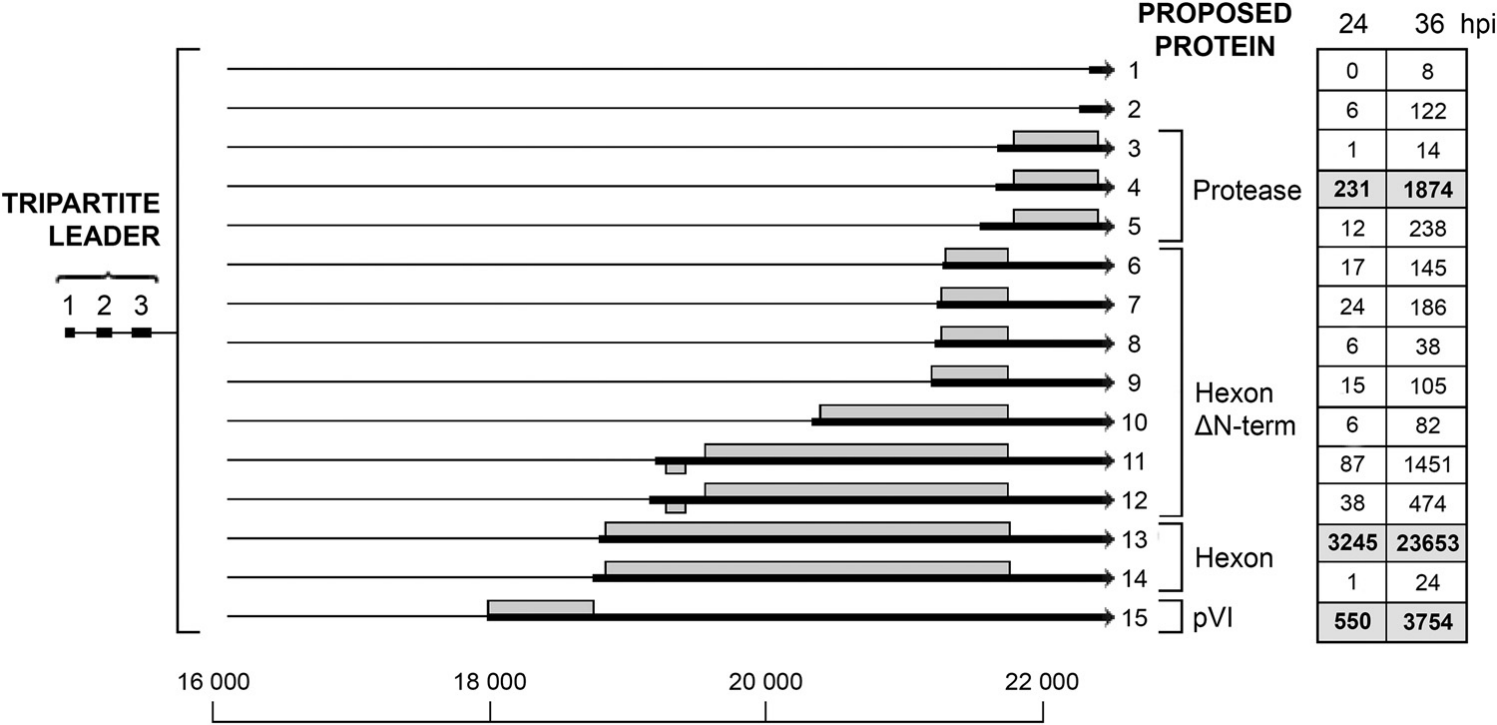
The L3 family of mRNAs. The figure shows the alternative spliced mRNAs detected from late region 3. The number of reads identified at 24 and 36 hpi are shown in boxes on the right, with previously described transcripts in bold in shaded boxes. Thick lines denotes exons, thin lines introns. Gray boxes indicate the proposed proteins expressed from respective mRNA. For a complete summary of the triple-verified L3 mRNAs, see Table S1.

The L3 unit encodes three well-known proteins, the structural hexon, pVI proteins, and the L3-23K protease (Fig. 10) (reviewed in reference 10). The previously characterized mRNAs encoding the three L3 proteins were all abundant in our Nanopore data. However, we also observed an unexpectedly high complexity of 3′ splice site usage within the L3 unit. As shown in Fig. 10, a low abundance of 12 alternative 3′ splice sites are used in the L3 region. Seven of these novel 3′ splice sites are likely to encode amino-terminal deletion mutants of the hexon protein (transcripts 6 to 12). With regard to this point, it is interesting that it was previously reported that hexons with different electrophoretic mobilities are present in Ad2-infected cells (24). Transcripts 11 and 12 are especially interesting, first, because they are fairly abundant, almost approaching the abundance of the protease mRNA (Fig. 10), and second, because they have an upstream reading frame with the capacity to encode a novel 5.6K protein. The initiator AUG for this predicted protein appears to be in a favorable configuration for being used in translation (see Discussion). Two novel 3′ splice sites producing alternative low-abundance mRNAs for the L3-23K protease (transcripts 3 and 5) and two 3′ splice sites that produce spliced RNAs, which most likely are noncoding RNAs (transcripts 1 and 2), were also identified.

### The L4 family of mRNAs.

The L4 unit encodes the minor pVIII cement protein and three regulatory proteins. The L4-100K protein has an important function as a specificity factor, enhancing the tripartite leader-dependent translation at late times of infection. The L4-22K and L4-33K proteins are important in the activation of MLP transcription and MLTU alternative RNA splicing, and also appears to have a function in viral DNA packaging (reviewed in references 10 and 25).

**FIG 11:**
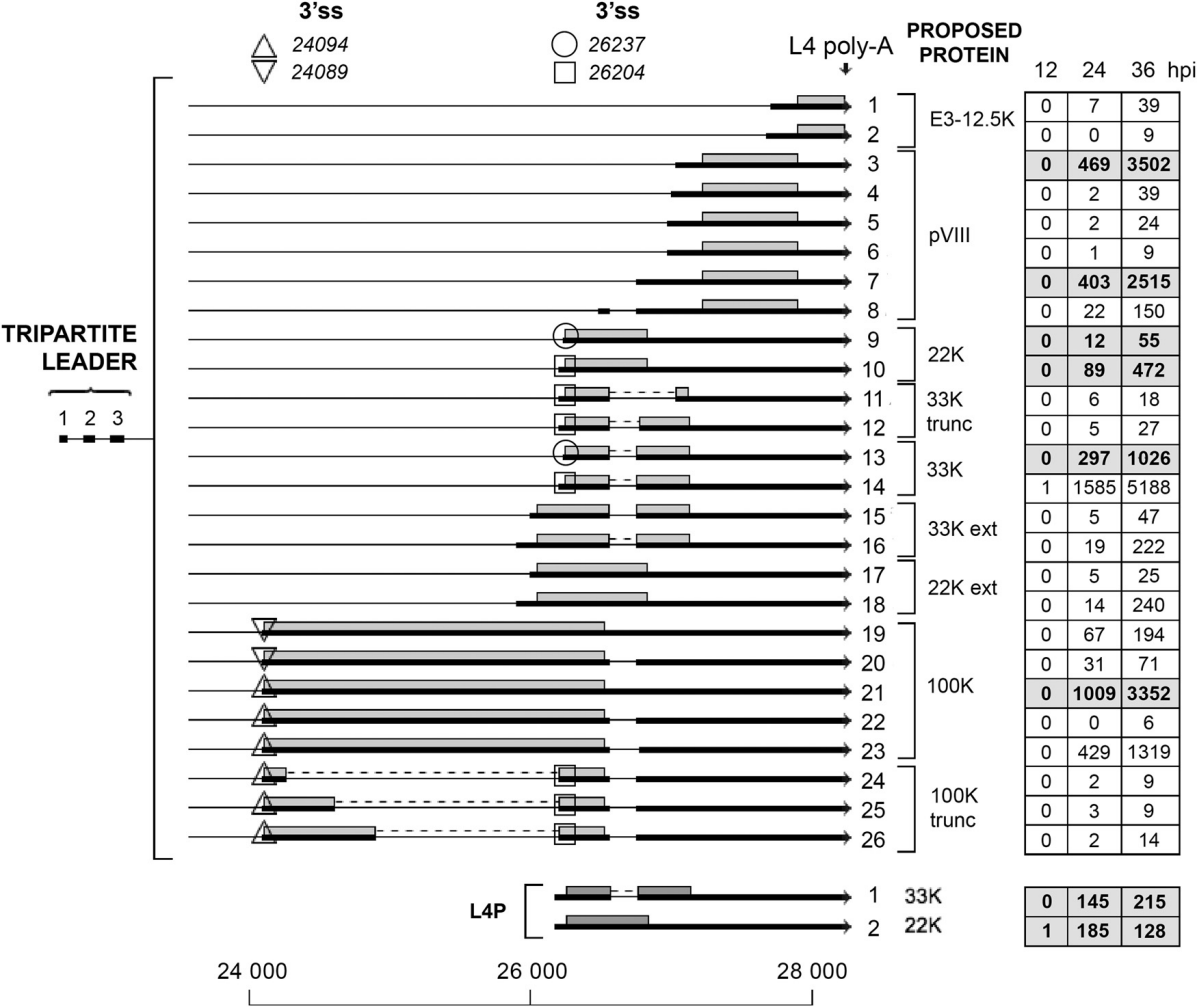
The L4 family of mRNAs. Alternative spliced mRNAs detected from late region 4 are shown. The numbers of reads identified at 12, 24, and 36 hpi are shown in boxes on the right, with previously described transcripts in bold in shaded boxes. Thick lines denotes exons, thin lines introns. Triangles (up or down) show microheterogeneities in 100K 3′ splice site usage. Circles and squares similarly show heterogeneities in 22K and 33K 3′ splice site usage. Gray boxes indicate the proposed proteins expressed from the respective mRNA. Poly-A, polyadenylation site. For a complete summary of the triple-verified L4 mRNAs, see Table S1.

As shown in Fig. 11, the 100K mRNA comes in five variants (transcripts 19 to 23) with two alternative 3′ splice sites connecting to the tripartite leader: the major 3′ splice site at position 24094 (transcripts 21 to 23) and a minor 3′ splice site located four nucleotides downstream (transcripts 19 and 20). In addition, we observed a low frequency of 100K mRNAs, encoding in-frame truncated 100K proteins (transcripts 24 to 26). Interestingly, because of alternative 3′ splice site usage, we also observed a low frequency of mRNAs that would be predicted to encode L4-22K and L4-33K proteins with a 65-amino-acid amino-terminal extension (transcripts 15 to 18). Since the L4 and E3 units partly overlap, we also observed two late mRNAs that would be predicted to encode the E3-12.4K protein (transcripts 1 and 2).

The recently discovered L4 promoter, which drives expression of the L4-22K and L4-33K mRNAs at an intermediate time of infection (7), was also detected (Fig. 11).

**FIG 12:**
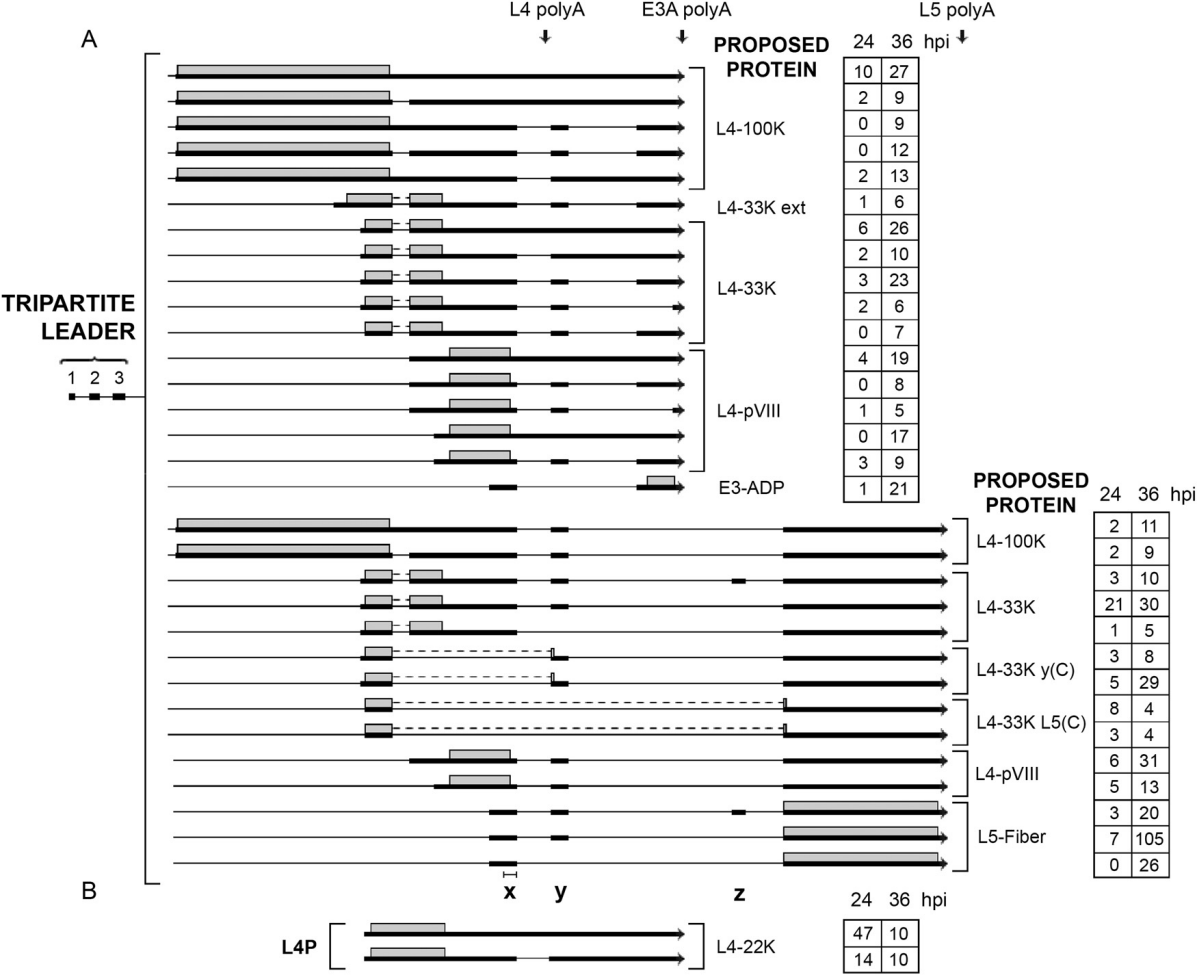
L4-E3-L5 readthrough mRNAs. (A) A novel class of mRNAs that we detected are L4 spliced mRNAs that bypass the normal L4 polyadenylation site and use the downstream E3A or L5 poly(A) sites. (B) Novel mRNAs initiated at the L4 promoter. The number of reads identified at 24 and 36 hpi are shown in boxes on the right. Thick lines denotes exons, thin lines introns. Gray boxes indicate the proposed proteins expressed from respective mRNA. The locations of the x, y, and z leaders are depicted at the bottom. The bar above the x leader shows the normal size of the x leader. Poly-A, polyadenylation site. For a complete summary of the triple-verified L4-E3-L5 mRNAs, see Table S1.

A series of L4 readthrough transcripts were also discovered (Fig. 12), where the normal L4 poly(A) site was skipped, and mRNAs instead used the E3A or L5 poly(A) sites. These mRNAs are all predicted to encode the normal L4 proteins except for four mRNAs, where the first L4-33K exon is spliced to the y-leader or the fiber 3′ splice site [L4-33K y(C) and L4-33K L5(C); Fig. 12A]. We also detected two mRNAs that initiated transcription from the L4 promoter, with an alternative usage of the E3A poly(A) site (Fig. 12B). This high complexity of alternative poly(A) site usage and alternative RNA splicing in the formation of L4 mRNAs is even further illuminated when we look at the production of E3 L5 mRNAs. Note that the E3-ADP mRNA and the L5-fiber mRNAs depicted in Fig. 12 are grouped as L4 mRNAs, instead of E3 mRNAs, since the x leader is extended using an alternative upstream 3′ splice site.

### The remarkable complexity of L4, E3, and L5 mRNAs expressed late after infection.

**FIG 13:**
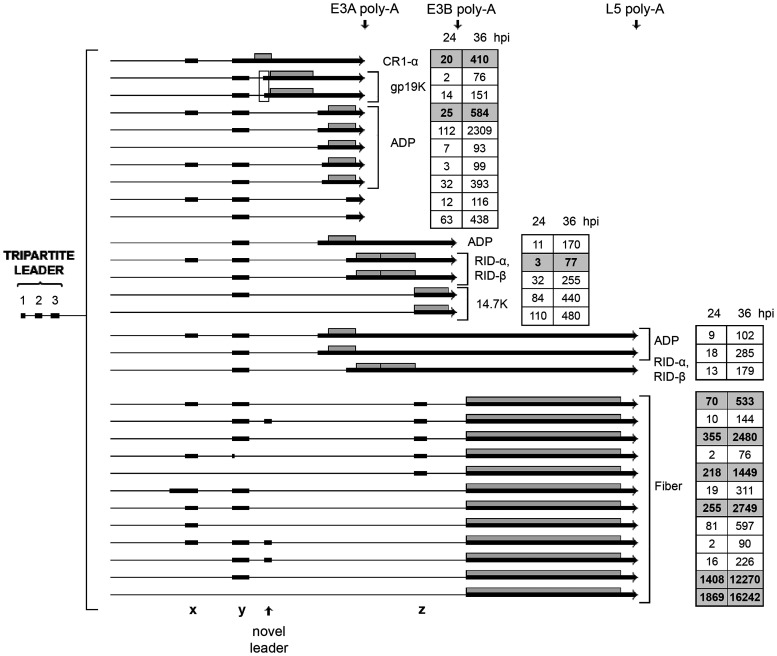
The complex splicing pattern of the E3 and L5 family of mRNAs expressed from the major late promoter at late times of infection. The number of reads identified at 24 and 36 hpi are shown in boxes on the right, with previously described transcripts in bold in shaded boxes. The boxed area shows two gp19K mRNAs that use two alternative 3′ splice sites separated by 16 nucleotides. Thick lines denotes exons, thin lines introns. Gray boxes indicate the proposed proteins expressed from respective mRNA. Poly-A, polyadenylation site. For a complete summary of the triple-verified L4-E3-L5 mRNAs, see Table S1.

At late times of infection, the E3 region produces mRNAs that are under the transcriptional control of the MLP. Since the L5 unit includes the x, y, and z leaders, there is a large combination of L5 mRNAs expressed (Fig. 13). L5 encodes the fiber protein, which makes up the receptor-binding antenna-like structure that protrudes from the vertex of the capsid (reviewed in reference 10). Since a subclass of the L4 mRNAs are also spliced to E3 exons and the L5 fiber exon (Fig. 12), the combined mRNA profile from the E3 region generates a bizarre number of alternatively spliced mRNAs (Table S1). Note that in Fig. 13 we used a cutoff of 75 to reduce the number of alternatively spliced mRNAs presented in the figure. In total, we detected more than 450 alternative spliced mRNAs from this region at 36 hpi (Table S1). This is by far the most complex splicing menagerie that we detect in the Ad2 transcriptome. Many of these were detected at a very low level, but all have been verified by our triple-verification strategy.

**FIG 14:**
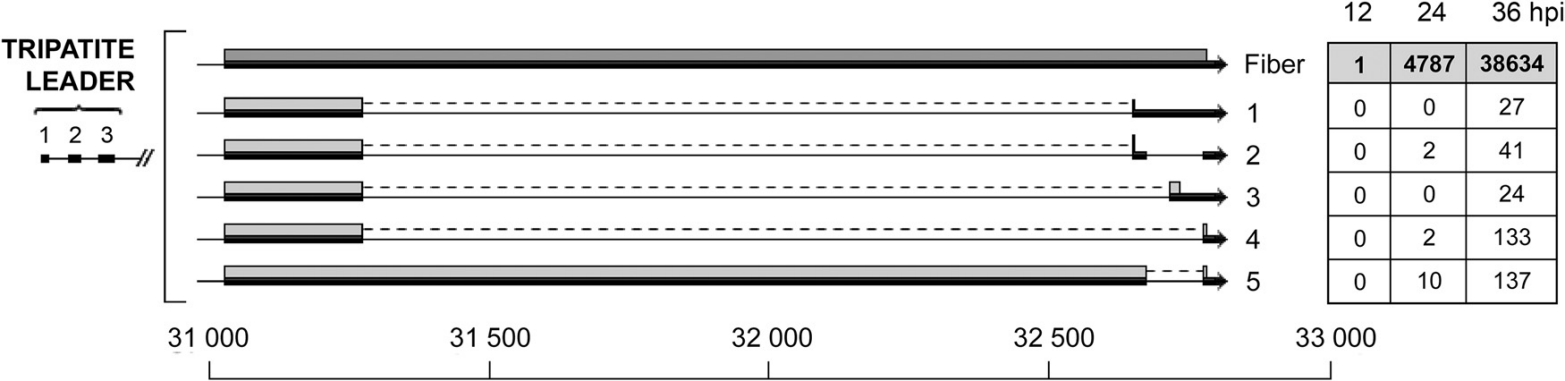
Alternative splicing events interrupting the fiber coding sequence. The numbers of reads identified at 12, 24, and 36 hpi are shown in boxes on the right, with the previously described fiber mRNA in bold in a shaded box. Thick lines denotes exons, thin lines introns. Gray boxes indicate the proposed proteins expressed from respective mRNA. For a complete summary of the triple-verified fiber alternative splicing events, see Table S1.

In addition, we detect a low number of fiber mRNAs containing an intron within the coding sequence. Three of them are in-frame deletions generating shortened fiber proteins (transcripts 4 and 5; Fig. 14), whereas three are out-of-frame deletions (transcripts 1, 2, and 3; Fig. 14).

## DISCUSSION

Here, we used the Oxford Nanopore Technologies long-read platform to characterize the structure of mRNAs expressed during an Ad2 infection. The majority of the most abundant early and late mRNAs had already been detected in the early electron microscopic studies by Chow and collaborators (3, 4). However, the present study reveals a much greater complexity of alternative RNA splicing with small variations in 5′ UTR splice site usage, producing multiple mRNAs encoding the same protein. A Nanopore analysis of the Ad5 transcriptome was recently published (26). Together with that study, our results demonstrate a significantly more complex splicing architecture for adenovirus than previously thought. The Ad5 study showed precise data on the complexity of alternative splicing, particularly within the E2, E3, and E4 regions. Most transcripts were found in both studies, but there are differences. However, we present a more complete picture of the complexity of adenovirus splicing. In total, we present data for more than 900 alternatively spliced mRNAs from the Ad2 transcriptome, more than 850 of which represent novel alternative spliced mRNAs. The most amazing complexity of splicing was observed in the L4, E3, and L5 mRNA cassettes, where a seemingly limitless variety of combinatorial alternative splicing events were detected (Fig. 12 and 13; Table S1). In total, we recorded more than 400 alternatively spliced mRNAs from this region alone. We also observed triple-verified hybrid mRNAs from the L1 to L3 families of mRNAs (Table S1), although these were found in significantly lower numbers than the L4, E3, and L5 mRNA cassettes.

An astonishing finding was that more than 50% of the total mRNAs from the E1A region initiated transcription upstream of the canonical E1A transcriptional start site. These novel potential transcriptional start sites mapped to a region in the viral DNA packaging repeat region and close to the ITR. Interestingly, the DNA packaging repeat region is known to bind cellular transcription factors (reviewed in reference 27) that are important for genome packaging. The region also functions as an E1A enhancer region and binds the cellular E2F and EF-1A transcription factors (28). Similarly, the ITR also binds cellular transcription factors, like NF1, Oct-1, SP1, and ATF (29). More recent work has shown that cellular enhancers and promoters are more similar than previously thought (reviewed in reference 30). Thus, enhancer transcription, or eRNA transcription, appears to be widespread in nature, with most active enhancers driving local eRNA transcription, using the same transcriptional machinery as promoters. The ITR region and the DNA packaging domain may contain novel promoters that remains to be discovered. Alternatively, transcription from or close to the inverted terminal repeat region and within the DNA packaging domain may cause eRNA synthesis that accounts for the novel 5′ extended E1A mRNAs that we detected (Fig. 2B). The existence of E1A mRNAs initiating upstream of the characterized E1A transcriptional start site has previously been observed *in vivo* (31, 32). As the ITRs are present at both ends of the viral genome, we propose that the 5′ extended E4 mRNAs in Fig. 5 (transcripts 6 to 9) may similarly result from transcription from a novel ITR promoter or alternatively be the result of eRNA transcription from or close to the inverted terminal repeat at the right-hand end of the viral genome.

Translation initiation is a key step in the regulation of eukaryotic gene expression. Dependent on the sequence context, the initiation process may start at multiple alternative start sites, including AUG and non-AUG start codons. This makes it challenging to predict the translational reading frame in the novel mRNAs that we detect in our revised Ad2 transcriptome. Eukaryotic mRNAs are typically functionally monocistronic, with the cap-proximal reading frame being used for translation. In addition, the Kozak rule (reviewed in reference 33) predicts the optimal sequence context around the initiator AUG codon for translation initiation. A caveat is that all adenovirus mRNAs are not functionally monocistronic. The classic example is the E1B 22S mRNA that encodes both the E1B-19K and E1B-55K proteins (34). Our analysis does not identify a separate mRNA encoding the E1B-55K protein, which agrees with the previous conclusion that the E1B-22S mRNA is functionally polycistronic (34). The E1B-19K reading frame is present in essentially all alternatively spliced E1B mRNAs (Fig. 2), making it the major E1B protein expressed. A further complication of the prediction of protein expression is that translation of the MLTU mRNAs uses a shunting mechanism for initiation of translation (20, 35). This mechanism allows the ribosome to bypass, or shunt, large regions of a 5′ UTR, even if a start codon in a good Kozak sequence context is bypassed. Thus, predicting the protein(s) that are expressed from an mRNA becomes highly uncertain. High-resolution mass spectrometry techniques have been used to study the Ad2 proteome (15). A re-evaluation of these data identified one peptide (MLQVWR) which corresponds to a protein initiating translation immediately downstream of the ITR region. Most likely, transcript 2 (Fig. 2B) is the novel spliced E1A mRNA connecting the identified amino terminus to the carboxy-terminal exon of the E1A 13S and 12S mRNAs. The identification of this peptide demonstrates that transcription from or close to the left ITR is active, resulting in mRNAs that are translated to novel proteins. Clearly, additional direct experimental evidence will be required to identify additional novel peptides and resolve the relationship between the transcriptome and the proteome.

The GT-AG consensus sequences at the 5′ and 3′ splice sites were remarkably conserved in the Ad2 transcriptome. Only two splicing events, which passed our triple-verification strategy, deviated from the GT-AG consensus rule. In the L4-100K reading frame, a GC-AG intron (nucleotides 24330 to 26204) would produce an in-frame shortened 100K protein (Table S1), adding to the repertoire of 100K truncated proteins (Fig. 11, transcripts 24 to 26). The second example was the 5′ splice site at the novel exon 2 in the E2A transcription unit, where we observed a microheterogeneity at the 5′ splice site, with approximately 2% of the splicing events using a noncanonical GC-AG splicing event (Fig. 3C). It is known that variants of the standard GT-AG intron exist in mammalian splicing. The major splice variant is the GC-AG intron, which accounts for an estimated 0.6% of annotated 5′ splice sites (36). It is possible that part of the bioinformatic approach we used to verify splice sites with the Nanopore and Illumina data is more efficient in identifying classical splicing events, thereby underestimating the number of splicing events that break the GT-AG rule. Most likely, a future analysis of the adenovirus transcriptome will produce additional infrequent splicing events breaking the classical GT-AG rule.

It is well established that adenovirus alternative splicing undergoes a regulated temporal shift in splice site usage (reviewed in reference 37). The typical examples that are often referred to are the shifts in E1A (Fig. 2) and L1 (Fig. 8) alternative splicing that produces the 9S mRNA and IIIa mRNAs, respectively, preferentially at the late stage of infection (reviewed in reference 38). However, this temporal shift in splice site usage is not unique to these units and can be seen in all the viral transcription units (Fig. 2 and 14). The general tendency is that shorter mRNAs, with larger introns spliced out, accumulate at the later time points of infection. The mechanistic details of this regulation have been most thoroughly studied for the E1A and L1 units (reviewed in reference 38). The results from these studies show that adenovirus modulates the activity of the cellular SR family of splicing factors (reviewed in reference 38) and encodes several proteins that have an effect on RNA splice site choice: E4-ORF3 and E4-ORF6 (39), E4-ORF4 (40), L4-33K and L4-22K (41, 42). It is not unreasonable to suspect that a viral remodeling, or relaxation, of the cellular spliceosome machinery may be part of the explanation for the production of the monumental number of alternatively spliced mRNAs observed during a lytic Ad2 infection.

An outstanding question is what roles the menagerie of novel RNAs play or whether they are spurious molecules generated by an overloaded splicing machinery. It is reassuring that cellular alternative RNA splicing also is highly complex (see for example reference 43) and that the paper by Donovan-Banfield et al. (26), which appeared while our manuscript was in preparation, shows that splicing in Ad5-infected cells is equally complex, although the overlap is incomplete. Thus, we are not dealing with technical artifacts or components which are unique to one experimental system. By using a limited number of promoters and poly(A) sites in combination with a large number of splice sites, the virus has decreased its evolutionary constraints. The use of alternative RNA splicing to expand the coding capacity of a genome is common both in cells and their viruses. It is likely that the plasticity in alternative RNA splicing enables the virus to fine-tune protein synthesis by providing different alternatively spliced mRNAs encoding the same protein under changing conditions. Also, the capacity to produce novel exon combinations will offer the virus an evolutionary advantage to change the gene expression repertoire and protein production in a changing environment. We cannot rule out the possibility that some of the novel transcripts identified are not translated into proteins. The tools to predict translation initiation events are imprecise, especially in virus-infected cells, leaving the possibility open that some of the transcripts we observed are in fact long noncoding RNAs (lncRNAs). Similar to the adenovirus genome, the human genome is subject to a pervasive transcription, with the vast majority of the genome being expressed as lncRNA. In humans, lncRNAs play an important role, functioning as scaffolds and in controlling gene expression in multiple biological processes, like transcription, RNA processing, and mRNA translation (reviewed in reference 44). Similarly, potential adenovirus-encoded lncRNAs may fine-tune viral gene expression at different stages of the replication cycle.

## MATERIALS AND METHODS

### Cell culture and Ad2 infection.

Human primary lung fibroblasts (IMR-90) purchased from American Type Culture Collection (ATCC) were cultured in Eagle's minimum essential medium (EMEM) supplemented with 10% fetal bovine serum and 1% penicillin/streptomycin (PEST) at 37°C in 5% CO_2_.

Cells were infected with Ad2 at a multiplicity of 100 fluorescence-forming units (FFU) per cell (titrated on HeLa cells) in serum-free medium (45). After 1 h adsorption at 37°C in 5% CO_2_, the medium was replaced with complete EMEM containing 10% FBS and incubated as described above. Infected cells were collected at 12, 24, and 36 hpi.

### RNA extraction and Oxford Nanopore sequencing.

Cytoplasmic RNA was prepared by lysis with IsoB-NP-40 buffer (10 mM Tris-HCl [pH 7.9], 150 mM NaCl, 1.5 mM MgCl_2_, 1% NP-40), followed by two rounds of phenol-chloroform-isoamyl alcohol extraction and one extraction with chloroform-isoamyl alcohol (46). For Nanopore cDNA sequencing, 500 ng total cytoplasmic RNA from each of the three time points was used as input for the ONT PCR cDNA protocol. Reverse transcription and strand switching were done with SuperScript IV (Invitrogen) for 20 min at 42°C followed by 20 min at 50°C and 10 min at 80°C. Libraries were barcoded and amplified with LongAmp for 14 cycles with a 6-min elongation time. A one-pot LSK-108 library (https://www.protocols.io/view/one-pot-ligation-protocol-for-oxford-nanopore-libr-k9acz2e) was prepared from the pooled cDNA. The resulting library was sequenced on a FLO-MIN106 flow using a MinION sequencing device. Raw sequencing data were base called using the Guppy base caller (Oxford Nanopore Technologies). For Nanopore direct RNA sequencing, 500 ng poly(A) selected RNA was prepared from the total cytoplasmic RNA prepared at 24 and 36 hpi. An ONT library was generated by following the protocol “Direct RNA Sequencing Protocol for the MinION” (SQK-RNA001), available on the Oxford Nanopore website, according to the manufacturer’s instructions. RNA strands from the resulting adapter containing RNA/cDNA hybrid library were sequenced on a FLO-MIN106 flow using a MinION sequencing device. The raw sequencing data were base called using the Guppy base caller (Oxford Nanopore Technologies) with the direct RNA base calling algorithm.

Computational analysis of sequencing data. cDNA reads produced by ONT sequencing were first selected depending on the completeness of the transcripts and then demultiplexed. The tool pychopper v0.6.9 (https://github.com/nanoporetech/pychopper) was used to identify full-length cDNA reads to select complete transcripts. In the demultiplexing step, first the updated and still supported qcat v1.0.1 (https://github.com/nanoporetech/qcat) was used. Unfortunately, qcat was not yet able to perform adapter trimming, so the unsupported tool porechop v0.2.4 (https://github.com/rrwick/Porechop) was used to trim adapters.

RNA direct fast5 files were base called using Guppy (version 3.0.3 + 7e7b7d0) 6 to trim the adapters for RNA while base calling. Then nanopolish (47), minimap2 (48), and SAMtools (49) were used to estimate poly(A) tail length and quality of the reads in order to select complete poly(A) tails (see https://nanopolish.readthedocs.io/en/latest/quickstart_polya.html for details of the process). The nanopore reads were aligned to HG38 (GRCh38) combined with the adenovirus 2 complete genome (J01917.1) with minimap2 (48). For practical reasons, the adenovirus alignment was then separated from the HG38 and analyzed alone. For the 12-hpi cDNA sequencing, 29,283 sequences aligned to the adenovirus genome. For the 24- and 36-hpi cDNA sequencing, the corresponding numbers were 108,273 and 285,809. For direct RNA sequencing, the numbers of adenovirus aligned reads were 20,711 and 33,102 for the 24- and 36-hpi experiments. All alternatively spliced mRNAs presented here were triple verified: i.e., they were identified by Nanopore cDNA sequencing (12, 24, or 36 hpi) and by Nanopore direct RNA poly(A)^+^ sequencing (24 and 36 hpi), and the splice junctions were verified using the Illumina data (12, 24, or 36 hpi, as described below). Transcript variants were manually identified by examining the minimap2 alignments of the cDNA and RNA direct Nanopore reads to the adenovirus genome in the Integrated Genome Viewer (IGV) (50) and by examining the table of alignment coordinates (described below). Individual splice junctions were considered verified if they were identified by TopHat (51), as previously described (19), and if the sequence reads in the HiSeq Illumina data (SRA accession no. SRX451094 to SRX451100) contained at least one read with a perfect k-mer spanning 9 nucleotides upstream and 9 nucleotides downstream of the junction. The k-mer test was made with a custom perl script scanning all reads in the forward and reverse directions and counting the reads having perfect matches to the 9+9 k-mers. The alignment coordinates were also extracted from the CIGAR strings in the SAM alignments and output into a table of aligned regions, reporting the coordinates of all alignment gaps equal to or larger than 20 nucleotides. This table was used to support identification of and quantify transcript variants. During transcript variant identification, the start and end of the alignments were allowed to deviate by 50 nucleotides from the expected start/end sites. When the start site was uncertain or was close to another start site or to a splice junction, this margin was adjusted appropriately. We also resequenced the Ad2 virus genome used in these experiments to eliminate artifacts by mutations in the k-mer analysis. The resequencing was done on an Illumina MiSeq machine using the Nextera XT library preparation kit. All splice junctions were compared to the mutations identified. None of the examined splice junctions were affected by the identified mutations.

### Mass spectrometry-based proteomics for verification of new transcripts.

Raw data from our previously published work (15) was used for confirming the existence of suggested transcripts also on the peptide/protein level. These data were produced using nano-liquid chromatography and high resolving mass spectrometry, and the full data set is available via the ProteomeXchange Consortium via the PRIDE (52) partner repository with the identifier PXD008980. Files from 36 hpi were processed in MaxQuant (version 1.6.5.0) (53). Database searches were performed using the implemented Andromega search engine (54) against a FASTA file consisting of 72 entries of potentially novel Ad2 peptides, predicted by the Nanopore sequencing data. False discovery rate (FDR) was calculated based on reversed sequences from the target-decoy search. An FDR of 1% was accepted for a match. Due to the likelihood of novel short peptides, peptides with four or more amino acids were considered, and a maximum of two cleavages were accepted. The mass tolerance was 4.5 ppm for the main search and 20 ppm for the fragment masses. Other standard settings were: trypsin as digesting enzyme, carbamidomethylation of cysteines as fixed modifications, and oxidation of methionine and acetylation of the protein N terminus as variable modifications. Peptides with matches were subjected to a BLAST search to control an Ad2 origin.

### Data availability.

The Nanopore cDNA and direct RNA sequencing data have been deposited in the Sequence Read Archive (SRA) and are available via BioProject no. PRJNA678228. The SRA accession numbers are SRR13052884, SRR13052885, SRR13052886, SRR13052887, and SRR13052888.

## Supplementary Material

Supplemental file 1
